# p53 in Canine Mammary Tumors: From Molecular Pathogenesis to Biomarker Applications and Comparative Oncology

**DOI:** 10.3390/vetsci13090984

**Published:** 2026-09-18

**Authors:** Ziqiao Wang, Yi Qin, Yongping Zeng, Shan Liao, Xiaoqi Tao, Xiao Liu

**Affiliations:** 1 College of Veterinary Medicine, Southwest University, Chongqing 400715, China; 19825661075@163.com (Z.W.); 13737707617@163.com (Y.Q.);; 2 Beagle Explore (Chengdu) Technology Co., Ltd., Chengdu 610219, China; 3 Chongqing Academy of Animal Sciences, Chongqing 402460, China

**Keywords:** canine mammary tumor, *TP53*, p53, biomarker, human breast cancer model, tumor suppression, molecular regulation

## Abstract

Canine mammary tumors (CMTs) are common neoplasms in female dogs and serve as an important naturally occurring model for studying human breast cancer. The p53 protein plays an important role in CMT biology, with alterations in the p53 pathway implicated in tumor development, progression, and tumor microenvironment regulation. However, reported frequencies of *TP53* mutations and p53 expression vary among CMT studies, and their associations with malignancy are not uniform. Thus, p53 has potential as a biomarker for CMT, although its clinical applicability requires standardized assessment and further validation. Studies examining p53 in combination with other molecular markers, p53–miRNA interactions, and the role of p53 in chemoresistance further highlight its potential diagnostic, prognostic, and therapeutic relevance. This review synthesizes current evidence on *TP53*/p53 alterations, biological functions, and biomarker applications in CMT, with particular emphasis on evidence heterogeneity, methodological limitations, and species-specific differences relevant to comparative oncology.

## Simple Summary

Canine mammary tumors are common in female dogs and share some important features with breast cancer in women, making them useful for studying how mammary tumors develop and progress. However, findings about the tumor-suppressing protein p53 in dogs have been inconsistent, and it remains unclear how reliably p53 can be used to assess tumor behavior or guide treatment. This review summarizes current knowledge of how changes in the gene responsible for p53 production, changes in the amount or distribution of the p53 protein, and changes in related biological processes are associated with canine mammary tumors. We also examine the potential value of p53 as a marker for identifying tumors, predicting disease outcome, and understanding responses to treatment, including its relationships with other tumor markers, small regulatory molecules, and resistance to anticancer drugs. The available evidence indicates that p53 may have important diagnostic, prognostic, and therapeutic value, but reported findings vary among studies and should therefore be interpreted with caution. Canine mammary tumors can provide a valuable naturally occurring model for studying breast cancer, but similarities between dogs and humans should not be regarded as complete equivalence. A better understanding of both shared and species-specific features may improve the use of canine tumors in comparative cancer research and ultimately support the development of more effective approaches to cancer diagnosis and treatment.

## 1. Introduction

Canine mammary tumors (CMTs) represent the most common neoplasm in female dogs [1]. Globally, CMTs account for 50% to 70% of all neoplasms in intact female dogs, with overall incidence rates strongly influenced by regional spaying practices. Notably, the ratio of benign to malignant lesions is approximately 1:1 [2]. While the exact peak age of incidence varies across epidemiological studies, CMT predominantly affects middle-aged and older dogs, with the highest risk typically occurring between 8 and 11 years of age [3]. Furthermore, age at onset appears to correlate with tumor biological behavior: benign lesions tend to emerge earlier (frequently between 7 and 9 years of age), whereas malignant tumors become increasingly prevalent in dogs of advanced age [4,5,6,7,8].

Human breast cancer (HBC) is a common malignancy among women, linked to multifactorial etiologies and characterized by diverse histological subtypes and molecular classifications [9]. Comparative analyses between HBC and CMTs reveal substantial similarities in sex distribution [10]. Some studies have observed similar age distribution patterns between the two diseases [11,12], while another study has suggested potential differences in age-related incidence patterns between the two species, primarily in that the age-specific incidence rate in women tends to plateau after natural menopause, whereas in canines it continues to rise steadily [13]. These discrepancies may reflect differences in study populations, geographic regions, diagnostic criteria, or sample sizes. Therefore, no definitive conclusion can be drawn at present, and the precise role of age in the comparative study of these two diseases warrants further investigation through standardized analyses.

**Figure 1:**
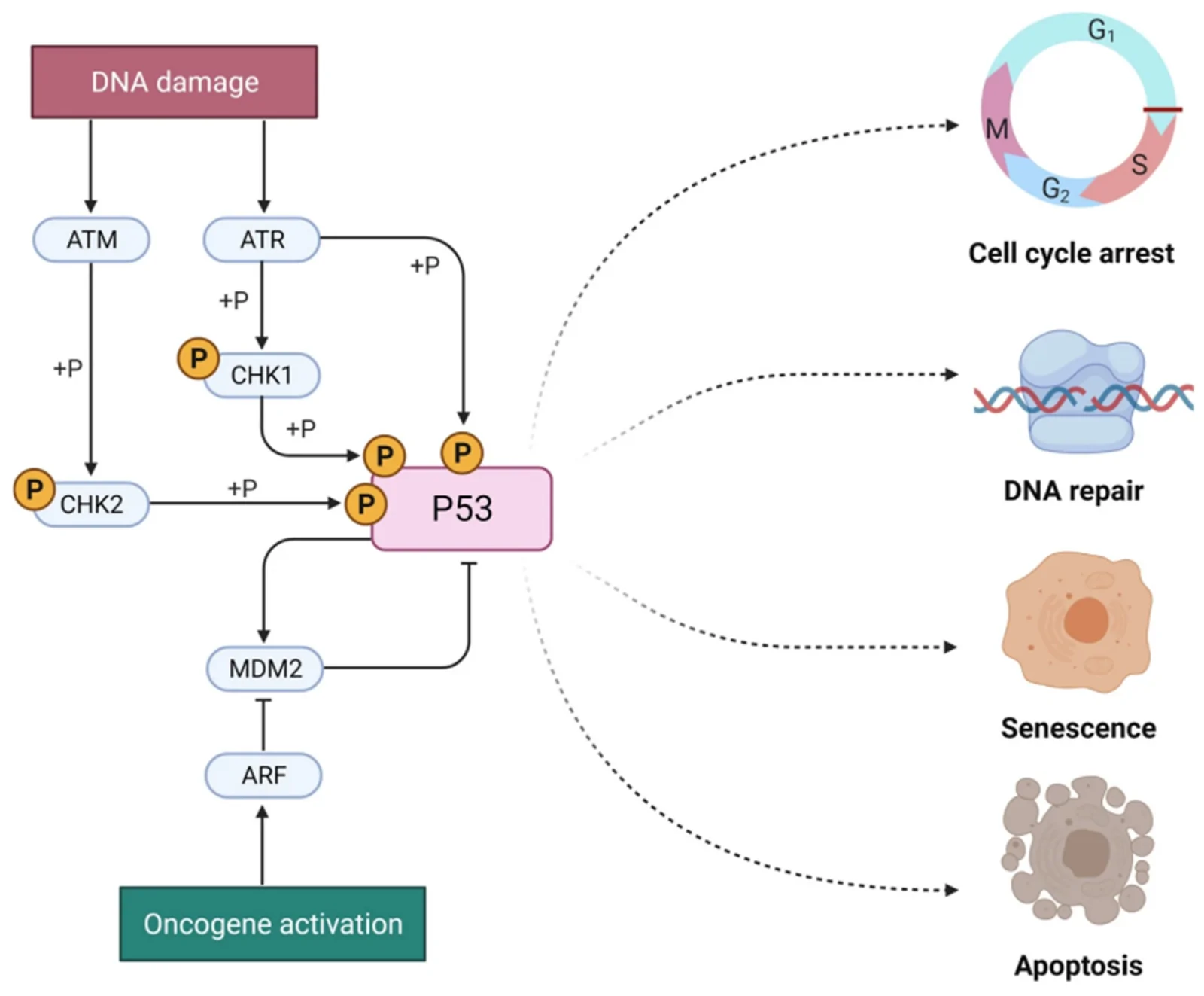
The DNA damage-induced p53 signaling pathway and its impact on cellular physiological processes. (Note: This figure was adapted from [17].).

The *TP53* gene is mutated in approximately half of human malignancies, including cancers of the breast, colon/rectum, lung, liver, prostate, bladder, and skin [14]. The p53 protein was first identified in 1979 as a host cellular factor complexed with the SV40 large T-antigen in transformed cells [15,16]. Encoded by the *TP53* gene on human chromosome 17, p53 functions primarily as a crucial tumor suppressor. As briefly illustrated in Figure 1, the p53 signaling network is activated by cellular stressors such as DNA damage (which triggers the ATM/ATR and CHK1/CHK2 kinase cascades) and oncogene activation (which stabilizes p53 via the ARF-MDM2 axis). Once activated, wild-type p53 orchestrates a multifaceted protective response: in addition to arresting the cell cycle to allow for DNA repair, it can also induce permanent cellular senescence or initiate apoptosis, thereby preventing the proliferation of genetically compromised cells and suppressing malignant transformation [17].

Given its critical role, evaluating p53 as a biomarker requires scientific precision. From the outset, it is essential to establish a clear conceptual distinction among three related but non-equivalent parameters: (1) *TP53* gene mutations, which represent specific sequence alterations at the genomic level [18]; (2) p53 protein expression or aberrant accumulation, which is typically evaluated at the tissue level using immunohistochemistry (IHC) [19]; and (3) functional alterations in the broader p53 pathway, which encompass disruptions in downstream effector signaling [17]. This conceptual distinction is critical for accurate data interpretation and cross-study comparison.

Similarly, p53 plays a pivotal role in the pathogenesis of CMTs. As early as 1996, van Leeuwen et al. demonstrated the critical involvement of p53 mutations in CMT development, underscoring its functional conservation across mammalian species, including humans, mice, and dogs [20]. Recently, advances in molecular diagnostics have further refined its evaluation: Brunetti et al. validated PAb240-based p53 IHC against next-generation sequencing (NGS) in canine tumors, establishing an optimal cutoff threshold of 10% positivity. This validation may improve the identification of CMTs with *TP53* alterations [21].

The objectives of this review are threefold: first, to critically summarize the current understanding of *TP53* mutation frequencies, p53 protein expression patterns, and functional consequences in CMTs; second, to evaluate the clinical and translational potential of p53 as a biomarker, both alone and in combination with other markers, in the context of diagnosis, prognosis, and treatment response; and third, to assess the strengths and limitations of CMT as a comparative model for p53-driven human breast cancer research, with explicit attention to evidence heterogeneity and species-specific differences.

This review was conducted by systematically searching PubMed and Web of Science databases for peer-reviewed literature published up to June 2026, focusing on canine mammary tumors and p53. The search strategy employed combinations of the following keywords using Boolean operators (AND/OR): “canine mammary tumor” or “canine mammary carcinoma,” each combined with “p53,” “*TP53* mutation,” “biomarker,” “prognosis,” “chemoresistance,” “microRNA,” and “comparative oncology.” Studies were included if they met the following criteria: (1) addressed p53-related mechanisms in canine mammary tumors or human breast cancer; (2) were published in peer-reviewed journals; (3) were original research articles or review papers; and (4) were written in English. Additionally, the reference lists of the included articles were manually screened to identify further relevant studies that may have been missed during the initial database search. Retrieved studies were first screened by title and abstract, followed by full-text assessment, and finally selected for discussion based on their direct relevance to the main themes of this review.

## 2. Canine Mammary Tumors as a Naturally Occurring Model for Human Breast Cancer

HBC remains the most commonly diagnosed cancer among women worldwide and a leading cause of cancer-related mortality in women [22].

CMTs are increasingly recognized as a valuable naturally occurring comparative model for HBC owing to their shared yet heterogeneous biological and molecular characteristics [23]. CMTs share several pathological and molecular features with HBC, including alterations in conserved cancer-related pathways; however, important species-specific differences also exist, and their value as a model does not depend on demonstrating complete equivalence between CMTs and HBC (Table 1) [23]. This cross-species conservation of selected biological pathways is particularly relevant when exploring the functional consequences and mechanistic pathways of p53 alterations, although similarities in p53 pathway function should not be interpreted as evidence of identical *TP53* mutation frequencies or mutation spectra across species. Unlike artificial animal models, CMT develop spontaneously under the combined influence of diverse environmental and genetic factors, thereby offering a disease etiology that more closely reflects the complexity of naturally occurring carcinogenesis while still requiring careful consideration of species-specific biological differences [24].

Previous studies have demonstrated that the *TP53* tumor suppressor gene plays a key role in tumorigenesis [28,29,30]. However, whether its expression patterns, genetic alterations, and functional consequences remain comparable across different species is still an open question. Kumaraguruparan et al. investigated 30 HBC patients and 30 female dogs with CMTs, utilizing IHC staining to analyze p53 expression in tumor tissues and adjacent normal tissues. Their results revealed that p53 expression was significantly increased in both HBC and CMTs compared to corresponding adjacent non-neoplastic tissues, whereas cytokeratin expression was markedly lower. Furthermore, the magnitude of these expression changes was more pronounced in premenopausal patients than in postmenopausal individuals [34]. These findings support involvement of the p53 pathway in mammary tumorigenesis across species; however, increased p53 protein expression should not be interpreted as direct evidence of *TP53* mutation, because p53 accumulation may also result from altered protein stability or other regulatory mechanisms [34].

Genomics and precision medicine have made remarkable strides in advancing human cancer treatment, yet their applicability across species remains to be fully defined [35]. Kim et al. conducted whole-exome and transcriptome analyses on 191 spontaneous CMTs cases displaying features relevant to HBC. They unveiled genomic features overlapping with those observed in HBC, notably *PIK3CA* mutations (43.1%), aberrations in the PI3K-Akt pathway (61.7%), and alterations in other key genes [26]. These findings support conservation of important oncogenic signaling pathways across species, but do not imply identical genomic profiles or mutation frequencies. Additionally, the study identified three distinct gene expression subtypes of CMTs, one of which shared strong similarities with the human basal-like HBC subtype, exhibiting an activated epithelial-to-mesenchymal transition (EMT), low claudin expression, and poor prognosis [26]. At the same time, the relative lack of *ERBB2* amplification and the absence of a clear HER2-enriched subtype in CMTs highlight species-specific molecular features [26]. Thus, canine molecular subtypes should not be considered direct equivalents of HBC subtypes, but rather as models of selected conserved molecular programs [26].

An increasing number of studies have focused on the molecular similarities between CMTs and HBC, particularly regarding cell cycle regulation and microRNA (miRNA) machinery. Despite originating from different species, CMTs and HBC display overlapping alterations across multiple genetic and molecular pathways rather than complete molecular identity [35]. Specifically, the *INK4a/ARF* locus is pivotal for cell cycle regulation, and its inactivation or mutation is commonly observed in various malignancies. Evidence shows that the expression pattern of *INK4a/ARF* in CMTs is associated with mammary tumor initiation and progression in a manner consistent with its established role in human tumorigenesis [32]. Furthermore, cancer-specific progression expression profiles (PEPs) in HBC correlate tightly with overall survival and prognosis in CMTs, reinforcing the validity of CMT as a model for HBC research [33]. Graim et al. performed RNA sequencing and mutation analysis on 89 mammary tissue samples (26 normal, 41 benign, and 22 malignant) from 16 dogs and developed the FREYA analytical framework. Their findings demonstrated that CMTs resemble human tumors in molecular subtypes and cancer-related mutations, harboring human PAM50 molecular signatures. The analysis identified PEPs where cancer-specific PEP genes were associated with tumor aggressiveness and effectively predicted survival outcomes in HBC patients [31]. Together, these findings suggest conservation of selected transcriptional and molecular programs between CMTs and HBC, while also underscoring that cross-species molecular classifications are not interchangeable.

To evaluate the potential of CMT as a translational model for HBC, research teams have performed genomic sequencing and drug sensitivity profiling. Whole-genome sequencing of 21 malignant CMTs indicated that the mutational burden and several mutational signatures were lower or less diverse than those reported in HBC, with only one case exhibiting a *TP53* P268T mutation in the DNA-binding domain [27]. Gene-editing experiments further supported the tumor-suppressive role of *TP53*, showing that its knockout conferred a distinct survival advantage to cells [27]. In addition, drug sensitivity testing was conducted using CMT organoid models, focusing on Nutlin-3a, an inhibitor of the MDM2-p53 interaction [27]. MDM2 negatively regulates p53, and its overexpression inhibits p53 activity to promote tumor growth [36,37]. Nutlin-3a restores the tumor-suppressive function of p53 by blocking MDM2 binding [36]. Organoid experiments demonstrated that the *TP53*-mutated organoid line was resistant to Nutlin-3a, whereas the *TP53*-wild-type organoid lines tested were sensitive [27]. This result supports a role for p53 status in determining therapeutic responses in CMTs organoids and suggests a potential association between *TP53* mutation status and resistance to MDM2-p53 pathway inhibition [27]. Tumor cells harboring *TP53* mutations likely evade p53-dependent apoptotic pathways, thereby acquiring resistance to p53-targeted therapies—a finding that provides key insights into therapeutic strategies for *TP53*-mutated cancers [27]. Importantly, the *TP53* finding should be interpreted cautiously because the reported mutation frequency is based on a relatively small cohort of 21 malignant CMTs and cannot be generalized to all CMTs. Differences in reported *TP53* alteration frequencies across studies may reflect variation in sample size, histological classification, tumor grade and stage, metastatic status, genomic regions analyzed, sequencing or mutation-detection technologies, and study-population characteristics such as breed, age, and reproductive status. Therefore, the available evidence supports a conserved functional role of the p53 pathway across CMTs and HBC, but does not establish identical *TP53* mutation frequencies or mutation spectra between the two species.

Nonetheless, using CMT as a model presents certain limitations, including stringent ethical regulations on animal welfare, substantially higher experimental costs compared to mouse models, and discrepancies in treatment paradigms—CMTs are primarily managed via surgical resection, whereas HBC utilizes multimodal therapies tailored to molecular subtype and disease stage [23,25]. Strengthening collaboration between veterinary and human medical researchers will be essential to advance the effective application of canine tumor models in oncology and facilitate clinical translation [23,25]. Overall, CMTs should therefore be regarded not as exact replicas of HBC, but as naturally occurring comparative models that reproduce selected aspects of HBC biology.

## 3. p*53* Mutations and Canine Mammary Tumors

p53 represents a highly conserved tumor suppressor, often referred to as the “guardian of the genome,” playing a pivotal role in maintaining genomic stability and preventing malignant transformation [37]. Although direct *TP53* mutations are less frequent in CMT than in HBC, the functional dysregulation of the p53 pathway remains a critical event in canine mammary tumorigenesis. Under physiological conditions, p53 suppresses aberrant cell proliferation by regulating the cell cycle, promoting DNA damage repair, and inducing apoptosis [38]. However, *TP53* mutations disrupt these homeostatic mechanisms, allowing damaged cells to evade normal regulatory controls and initiate tumor formation [39]. Mutation-induced structural or functional alterations impair the ability of p53 to respond to DNA damage, thereby accelerating genomic instability—a process considered a central mechanism in CMT development [40]. Further studies, primarily in human and murine models, reveal that *TP53* mutations predominate within its DNA-binding domain; missense point mutations in these “hotspot regions” lead to a loss-of-function (LOF) or gain-of-function (GOF), weakening downstream gene regulation [41]. Consequently, mutant p53 directly or indirectly upregulates pro-tumorigenic genes, such as matrix metalloproteinases (MMPs) and vascular endothelial growth factor (VEGF), which drive cell invasion, migration, and angiogenesis [42]. These functional modifications demonstrate that p53 mutations are not merely “passive events” in tumorigenesis, but also play active roles in driving tumor invasion and metastasis.

**Figure 2:**
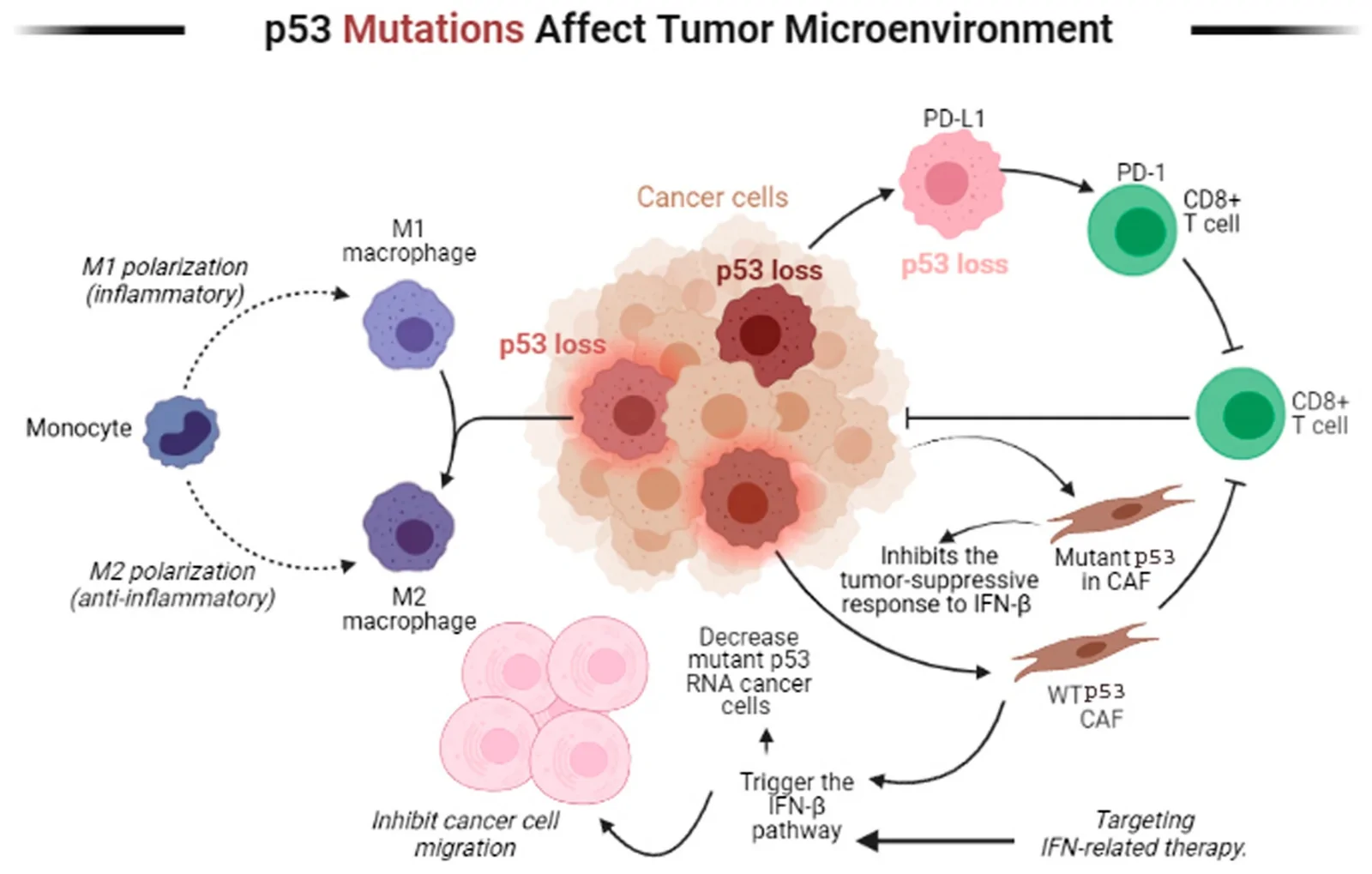
Immunomodulatory effects of mutant p53 and p53 loss on the tumor microenvironment. Legend: The schematic diagram illustrates how p53 mutations and loss in cancer cells profoundly reshape the tumor microenvironment (TME) to establish an immunosuppressive niche. (1) Macrophage Polarization: p53 alterations skew monocyte differentiation away from the pro-inflammatory, anti-tumor M1 macrophage phenotype and promote the anti-inflammatory, pro-tumorigenic M2 macrophage phenotype. (2) Immune Checkpoint Regulation: p53 deficiency induces the upregulation of PD-L1 on cancer cells, which binds to PD-1 on CD8+ T cells, thereby suppressing their cytotoxic effector functions and promoting immune evasion. (3) Crosstalk with CAFs: Mutant p53 interacts with CAFs to inhibit the tumor-suppressive response of the IFN-β pathway (which otherwise triggers a decrease in mutant p53 RNA in cancer cells and inhibits cancer cell migration). Conversely, targeting IFN-related therapy can reactivate this pathway. (Note: This figure was adapted from [17].).

Notably, the effects of p*53* mutations extend beyond intrinsic cellular signaling to profoundly reshape the tumor microenvironment (Figure 2) [17,43]. Evidence from human and murine cancers indicates that mutant p53 alters downstream transcriptional activation patterns, inducing the expression of pro-inflammatory and angiogenic factors to establish a favorable niche for tumor propagation [44]. Specifically, these alterations amplify local inflammatory responses and stimulate neovascularization, fueling tumor cells with essential nutrients and metastatic conduits that ultimately promote cancer progression. Investigating 16 distinct genetically engineered mouse models of breast cancer, Wellenstein et al. demonstrated that p53-deficient breast cancer cells secrete WNT ligands (such as WNT1, WNT6, and WNT7A) to activate tumor-associated macrophages (TAMs) to produce IL-1β, thereby triggering systemic inflammation [45]. This inflammatory cascade fuels the expansion and activation of neutrophils—particularly immature cKIT+ neutrophils—which subsequently drive breast cancer metastasis.

Another study utilizing human cancer cell models revealed that the secretion of mutant p53 relies on its N-terminal dileucine motif, facilitating exosome-mediated secretion through binding to β-adaptin, whereas CHK2-mediated phosphorylation at Ser20 inhibits this secretory process [46]. In murine in vivo experiments, mutant p53 significantly reduced the abundance of tumor-infiltrating CD4+ T lymphocytes and induced T-cell exhaustion by suppressing the expression of key glycolytic enzymes (such as p-PKM2, PFKP, and HK-I), thereby promoting tumor immune evasion [46]. Furthermore, inhibiting mutant p53 secretion—either by knocking down *AP1B1* or mutating the dileucine motif—partially restored CD4+ T lymphocyte counts and function while suppressing tumor growth [46]. These findings suggest that targeting and blocking the secretion of mutant p53 represents a promising therapeutic strategy for cancer [46]. As illustrated in Figure 2, the loss or mutation of p53 in cancer cells profoundly remodels the TME toward an immunosuppressive state. Beyond the aforementioned mechanisms, studies in human and murine models demonstrate that p53 loss skews monocyte polarization away from a pro-inflammatory M1 phenotype and promotes an anti-inflammatory M2 macrophage phenotype. Furthermore, p53 deficiency induces the upregulation of PD-L1 on cancer cells, which subsequently binds to PD-1 on CD8+ T cells, suppressing their cytotoxic effector functions. Additionally, cross-talk between mutant p53 in cancer cells and cancer-associated fibroblasts (CAFs) interferes with the IFN-β pathway, ultimately inhibiting tumor-suppressive responses [17,43]. It is important to explicitly note that these intricate immunomodulatory mechanisms have been elucidated almost entirely through human and murine studies. Currently, there is a lack of direct experimental data confirming these specific p53-TME interactions within CMTs. Nevertheless, given the strong cross-species conservation of the broader p53 signaling network, these non-canine models provide a vital comparative framework, highlighting critical knowledge gaps that warrant future veterinary oncology research. Loss-of-function p53 fails to arrest the cell cycle in response to DNA damage or induce apoptosis in aberrant cells, leading to heightened genomic instability and further accelerating tumorigenesis [47]. Collectively, these mechanisms underscore the central role of p*53* mutations in the initiation and progression of CMT.

## 4. p53 as a Biomarker for Canine Mammary Tumors

### 4.1. p53 Alterations and Malignancy in Canine Mammary Tumors

In CMTs, *TP53* mutations have been reported at variable frequencies across studies, and some studies have suggested an association with malignant features [48,49]. Muto et al. investigated p53 alterations in exons 5–8 using PCR followed by direct sequence analysis in 63 CMTs, identifying mutations in 4 of 38 benign tumors (11%) and 6 of 25 mammary carcinomas (20%) [48]. The authors found no significant relationship between p53 alterations and histological type or breed, although the higher frequency observed in carcinomas suggested a possible association with malignancy [48]. Lee and Kweon similarly analyzed exons 5–8 in 20 spontaneous CMTs using PCR and direct sequence analysis and detected p53 mutations in 40% of malignant tumors and 30% of benign tumors, corresponding to an overall frequency of 35% [49]. Taken together, these studies indicate that the reported frequency of *TP53* mutations in CMT is heterogeneous rather than uniformly high. Differences in sample size, histological composition, tumor grade or stage, breed and study population, genomic regions examined, and mutation-detection or sequencing methods may contribute to variation among studies [48,49]. Therefore, reported *TP53* mutation frequencies should be interpreted in the context of the specific cohort and methodological design rather than generalized to all CMTs.

Upon p53 inactivation or mutation, neoplastic cells may evade cell-cycle checkpoints, bypass apoptotic signals, and undergo sustained proliferation, thereby contributing to tumor progression [50]. In a large pan-canine cancer analysis, Alsaihati et al. reanalyzed whole-exome and whole-genome sequencing data from canine tumors of multiple tumor types and breeds and reported *TP53* mutations in 16.7% of 597 canine tumors overall [51]. Importantly, this figure represents the overall canine tumor cohort and should not be interpreted as a CMT-specific mutation frequency. The study nevertheless demonstrated an association between *TP53* mutation and tumor mutational burden (TMB) across canine tumor types and breeds, while CMTs were characterized by relatively infrequent *TP53* mutation and low TMB [51]. These findings provide broader evidence for a relationship between *TP53* alterations and genomic instability in canine cancer, but do not establish a specific *TP53* mutation frequency or a direct causal relationship with malignancy in CMTs. Moreover, evidence from CMTs and other canine tumor types suggests that alterations in *TP53* may occur together with changes in other oncogenic pathways, although the biological interaction between *TP53* and PI3K pathway alterations in CMTs requires further direct investigation [51]. Overall, the available evidence supports a role for *TP53* alterations in canine tumorigenesis, while highlighting substantial variation among studies and the need to interpret CMT-specific mutation frequencies cautiously.

While *TP53* mutations are recognized as important factors in the malignant progression of CMTs, the current literature suggests they may not typically serve as strictly independent prognostic indicators. In many clinical observations, p53 dysregulation appears to be associated with a spectrum of established aggressive clinicopathological features [52]. For instance, aberrant p53 protein accumulation and *TP53* mutations have been frequently linked to higher histological grades, increased mitotic activity, and elevated Ki-67 proliferation indices [53,54]. The frequency of these alterations also varies across morphological subtypes, generally being reported more often in simple, solid, and anaplastic carcinomas than in complex or mixed-type tumors [52,55]. From a comparative oncology standpoint, p53 dysfunction in CMTs is often observed alongside a reduction or loss of estrogen and progesterone receptors (ER/PR negativity). This biological pattern shares similarities with the hormone-independent nature of certain human basal-like or triple-negative breast cancers [56]. Because p53 status frequently overlaps with these other malignant features, its independent prognostic significance may diminish during multivariate survival analyses. When adjusting for clinical variables such as lymph node metastasis and overall clinical stage, p53 does not always maintain statistical significance [53,57]. Ultimately, rather than acting solely as a standalone prognosticator, *TP53* status may be better understood as a molecular indicator that reflects a highly proliferative and histologically advanced disease state.

### 4.2. Multi-Biomarker Panel Detection Involving p53

With advancements in molecular diagnostics, multiplex detection combining p53 with other biological markers has been investigated as a potential approach for the diagnosis and clinical assessment of CMTs [58]. In one study, serum and tissue expression patterns of VEGF, SF, p53, and NLRP3 were evaluated in healthy dogs and dogs with benign or malignant mammary tumors, with concordant trends observed between tissue and serum samples [58]. However, the biomarker analysis was performed in a relatively small cohort of 90 dogs, including 30 healthy dogs, 30 dogs with benign mammary tumors, and 30 dogs with malignant mammary tumors [58]. ROC analysis showed that the combined four-marker panel (VEGF + SF + p53 + NLRP3) achieved a sensitivity of 93.3% and an accuracy of 75.4%, but its specificity was only 55.6% [58]. These findings suggest that multiplex biomarker assessment may improve sensitivity for CMT detection, although the relatively small study population and limited specificity warrant further validation before clinical implementation.

Studies evaluating p53 and HER2 expression in CMTs have suggested that their combined assessment may provide additional information on tumor biological characteristics; however, the prognostic value of this combination remains insufficiently validated [59]. Furthermore, Brunetti et al. evaluated p53, ER, and Ki67 expression by IHC in 170 CMT cases [54]. p53 positivity was reported in 8/170 cases (4.7%), whereas ER was positive in 69/170 cases (40.5%), and the mean Ki67 index was 24% ± 18% [54]. The relatively low frequency of p53 positivity in this cohort should be interpreted cautiously and does not necessarily indicate a low frequency of *TP53* alterations in CMTs. Differences in immunohistochemical antibodies, staining and positivity criteria, cut-off values, histological characteristics of the tumors, and underlying molecular alterations may contribute to variation in reported p53 expression among studies. In particular, immunohistochemical p53 positivity reflects detectable protein expression or protein accumulation and should not be considered equivalent to *TP53* mutation status. In this study, p53 positivity was associated with higher Ki67 indices and higher histological grades, whereas ER positivity was associated with lower Ki67 indices, lower histological grades, and significantly longer survival [54]. These findings suggest that integrated assessment of p53, ER, and Ki67 may provide complementary information for evaluating the biological behavior of CMT, although the prognostic utility of this panel requires further validation in larger and independent cohorts.

Overall, while multi-biomarker panels have shown statistical associations with tumor characteristics, their clinical utility remains limited by the reliance on single, relatively small cohorts. Crucially, standardized IHC cut-off values and independent validation cohorts are needed to establish the reproducibility and clinical utility of these biomarker panels. Where feasible, cross-validation with NGS-based genomic or transcriptomic profiling could further clarify whether these protein-level biomarkers reflect underlying molecular alterations before such experimental panels are considered for routine clinical decision-making.

### 4.3. Interaction Between p53 and microRNAs

MicroRNAs (miRNAs) are small, endogenous non-coding RNAs that regulate post-transcriptional gene expression across diverse physiological and pathological processes [60]. Dysregulated miRNA expression is frequently associated with tumor cell proliferation, metastasis, and resistance to apoptosis [60]. The relationship between miRNAs and the p53 pathway is bidirectional, with p53 capable of regulating specific miRNAs and miRNAs modulating components of the p53 signaling network [61]. However, the extent to which these interactions contribute to CMT development remains incompletely defined.

Several studies have documented dysregulated miRNA expression in CMTs. Boggs et al. reported that CMTs exhibited miRNA expression patterns broadly resembling those observed in HBC including increased expression of miR-29b and miR-21, although miR-145 showed a different pattern [62]. Similarly, Kim et al. demonstrated by RT-qPCR that miR-21 and miR-10b were significantly upregulated in both benign and malignant CMTs, whereas miR-34a was downregulated [63]. These findings provide evidence of miRNA dysregulation directly in CMTs, but do not by themselves establish a functional interaction between these miRNAs and the p53 pathway.

miR-34a is of particular interest because it has been reported to be dysregulated in both CMTs and HBC, suggesting potential relevance to comparative mammary cancer research [64]. Bulkowska et al. subsequently characterized miRNA expression in canine mammary cancer and identified distinct miRNA expression patterns associated with tumor type, malignancy grade, and particularly metastatic status [65]. Although the regulatory relationship between p53 and miR-34a has been demonstrated in other experimental cancer models, in which p53 can induce miR-34a expression as part of a tumor-suppressive response [66], direct functional evidence demonstrating this regulatory axis in CMTs remains limited. More recently, a 2026 study reported increased miR-34a expression in malignant CMTs and identified miR-34a as a potential diagnostic biomarker for distinguishing malignant from benign mammary tumors [67]. These findings indicate that miR-34a is relevant to CMTs biology and may have diagnostic potential, while the specific functional relationship between miR-34a and p53 in CMTs remains to be clarified.

miR-34c is another member of the p53-regulated miRNA family and generally functions as a tumor suppressor [68]. In a study by Fish et al., serum miRNA profiling was conducted in 10 healthy female dogs and 10 dogs with histologically confirmed CMTs [69]. Using deep RNA sequencing and digital droplet PCR (dPCR), 452 unique serum miRNAs were identified, of which 65 showed significant differential expression (fold-change >± 1.5) [69]. Although miR-34c was upregulated, circulating miR-18a and miR-19b showed greater potential as diagnostic biomarkers of CMTs [69]. These circulating miRNA findings primarily represent biomarker associations and should be distinguished from direct evidence of p53–miRNA regulatory interactions in CMTs. In 2018, Fish et al. further demonstrated that malignant canine mammary epithelial cells release exosomes containing differentially expressed miRNAs that regulate oncogenic networks, supporting the potential utility of exosome-derived miRNAs for non-invasive molecular assessment of CMTs [70]. However, the clinical translation of circulating and exosomal miRNAs remains influenced by methodological factors, including sample collection and processing, RNA extraction, normalization strategies, and analytical platforms. Larger studies using standardized protocols and independent canine cohorts are needed to establish the reproducibility and clinical utility of these candidate biomarkers.

### 4.4. p53 and Chemoresistance

p53 plays an important role in the cellular response to chemotherapy by regulating cell-cycle checkpoints and apoptosis [71]. However, depending on the cellular context p53 signaling may also influence cell survival and repair following genotoxic stress, contributing to heterogeneous therapeutic responses [72]. The p53–telomerase reverse transcriptase (TERT) axis has also been implicated in telomere maintenance, oxidative stress regulation, and therapeutic resistance in several cancer models [73,74,75,76]. TERT activation has been reported to protect tumor cells from DNA-damaging agents and promote cellular survival [74]. These findings provide a mechanistic basis for considering a potential relationship between p53, telomere maintenance, and chemoresistance, although the relevance of this axis to CMTs remains to be further investigated [73,74,75,76].

In CMTs, experimental evidence suggests that p53-related signaling may influence responses to therapeutic stress. Levi et al. directly investigated the response of two CMT cell lines to doxorubicin (DOX) treatment [77]. In these CMT cell lines, DOX treatment induced p53 activation, cell-cycle arrest, and apoptosis [77]. Surviving cells following DOX exposure exhibited features associated with reduced treatment sensitivity, suggesting a potential role for p53-related responses in cellular adaptation to chemotherapy [77]. The study further suggested an association between p53 and TERT expression, telomere maintenance, and antioxidant capacity in the response of these CMT cell lines to DOX [77]. These findings provide direct experimental evidence from CMT cell lines, but their relevance to chemoresistance in naturally occurring CMT and canine patients remains to be determined.

Epithelial-to-mesenchymal transition (EMT) describes a process in which neoplastic cells lose epithelial characteristics, such as E-cadherin expression, and acquire mesenchymal features, including Vimentin expression, thereby enhancing migratory and invasive capacities [78]. Studies in human and other cancer models have indicated that p53 loss or mutation can influence TGF-β signaling and EMT-associated transcription factors, including Snail and ZEB1 [79]. Similarly, p53-related regulation of cancer stem cell (CSC) properties and the TME has been implicated in tumor progression and therapeutic resistance [80]. These observations provide a mechanistic framework for investigating whether comparable p53-associated EMT, CSC, and TME processes contribute to CMT progression and treatment resistance. A review of EMT in canine tumors indicates that EMT-related mechanisms have been investigated across canine neoplasms, although their specific contributions to p53-associated chemoresistance in CMTs require further clarification [81].

From a comparative oncology perspective, the utilization of CMT as a translational model for human breast cancer is supported by the high degree of genetic conservation within the *TP53* gene between canines and humans, including strong conservation of the *TP53* gene and core downstream p53 signaling pathways. However, important biological differences remain. The differing immune landscapes, varied tumor microenvironment dynamics, and the relative scarcity of extensive clinical trials evaluating p53-targeted therapies in canine patients represent important limitations when directly translating these in vitro mechanisms to human clinical outcomes.

Overall, current evidence supports a role for p53-related signaling in therapeutic responses of CMT cells, particularly based on doxorubicin-treated CMT cell-line studies [77]. In contrast, evidence concerning the p53-TERT axis, EMT, CSC properties, and associated microenvironmental mechanisms is derived from a combination of CMT studies and broader cancer research. These pathways therefore represent biologically plausible mechanisms for further investigation in CMTs, while their clinical relevance as determinants of chemoresistance in canine patients remains to be established.

## 5. Limitations of Current Evidence

Several limitations should be considered when interpreting the current evidence on p53 in CMTs. First, substantial heterogeneity exists among studies in sample size, breed, histological composition, genomic regions examined, detection methods, and criteria for p53 immunohistochemical assessment, limiting direct comparison of reported findings. Second, although CMT-specific studies provide evidence for the involvement of p53 in tumor biology, some mechanistic insights concerning the p53–TERT axis, EMT, cancer stem cells, tumor microenvironment, and chemoresistance are derived partly from other cancer models and therefore require further confirmation in CMT-specific systems. Third, much of the available evidence is based on retrospective cohorts, cell lines, organoid models, or relatively small study populations, while prospective clinical validation in canine patients remains limited. Finally, similarities between CMTs and HBC support their comparative value but do not imply complete biological equivalence, and species-specific differences should be considered when interpreting the translational relevance of p53-related findings. These limitations highlight the need for standardized methodologies, larger prospective cohorts, and independent validation before p53-related findings can be consistently translated into clinical applications.

## 6. Conclusions and Future Perspectives

This review summarizes current evidence on the involvement of the p53 pathway in CMT biology, including tumor progression, therapeutic response, biomarker development, and interactions with the tumor microenvironment. Available studies indicate that *TP53* alterations occur at variable frequencies across CMT cohorts, and their biological and clinical significance may differ according to tumor characteristics and methodological approaches. Although p53-related mechanisms have been associated with malignant phenotypes and therapeutic responses, the available evidence remains heterogeneous, and several proposed mechanisms require further validation in CMT-specific models.

Future research should focus on prospective studies involving larger and well-characterized cohorts to determine the clinical significance of *TP53* alterations and p53 expression. Particular attention should be given to breed-specific and histotype-specific mutation profiles and their relationships with tumor behavior and therapeutic response. Standardization of *TP53* mutation detection and p53 immunohistochemical assessment, including reproducible IHC cut-off values, is needed to facilitate comparison among studies. Independent validation cohorts will also be essential to establish the reproducibility and clinical utility of p53-related biomarkers before their incorporation into routine veterinary clinical practice.

Further investigation of gene-editing and precision medicine approaches may provide new opportunities for targeting p53-related alterations. In parallel, integrated multi-omics approaches could help define relationships among p53 alterations, tumor heterogeneity, and the tumor microenvironment. Together, these approaches may improve understanding of p53 biology in CMTs and help determine which findings have sufficient evidence for clinical translation.

## Figures and Tables

**Table 1:** Comparative clinicopathological, molecular, and genetic features of HBC and CMT.

Feature	HBC	CMT	Cross-Species Correspondence	References
Occurrence	Spontaneous	Spontaneous	Similar	[23,24]
Age at onset	Occurs predominantly in middle-aged and older women	Occurs predominantly in adult and older female dogs	Age-associated in both species, but chronological ages are not directly equivalent	[23,24]
Disease course	Heterogeneous and influenced by molecular subtype, histological grade, stage, and treatment	Heterogeneous and influenced by histological type, grade, stage, hormonal status, and other biological factors	Broadly comparable, but not identical	[23,25]
Tumor size	Variable at diagnosis and associated with disease stage and prognosis	Variable at diagnosis and associated with malignancy and prognosis	Comparable, but direct cross-species comparison requires caution	[23,25]
Clinical stage	TNM-based staging is widely used	Staging incorporates primary tumor characteristics, regional lymph node involvement, and distant metastasis	Conceptually comparable, but staging criteria differ	[23,25]
Lymph node involvement	Regional lymph node metastasis is an important component of staging and prognosis	Regional lymph node metastasis occurs in malignant CMTs and has prognostic relevance	Biologically comparable, but frequencies and patterns may differ	[23,25]
Common spontaneous mammary malignancy	Breast cancer is the most commonly diagnosed cancer among women worldwide	Mammary tumors are among the most common tumors in intact female dogs	Comparable within species-specific epidemiological contexts	[23,24]
Hormonal/reproductive influence	Hormonal and reproductive factors influence breast cancer risk	Ovarian hormonal exposure and reproductive history influence mammary tumor risk; early ovariectomy markedly reduces risk	Strong biological similarity with species-specific differences	[23,24]
Histological classification	Invasive breast carcinoma, particularly invasive carcinoma of no special type, is a major histological category	Carcinomas constitute a major malignant category, with substantial histological heterogeneity	Broadly comparable, but classification systems differ	[23,25]
Benign/precursor lesions	Benign and precursor lesions occur and may be associated with subsequent malignant transformation depending on lesion type	Benign mammary lesions and precursor-like alterations occur	Partially comparable; definitions and biological significance differ	[23,24,25]
Genetic and Molecular Alterations	Recurrent alterations include *PIK3CA*, *TP53*, *PTEN*, *AKT1*, and other cancer-related genes	Alterations in conserved cancer-related genes, including *PIK3CA*, *TP53,* and *AKT1*, have been reported	Partially conserved; mutation frequencies and spectra may differ	[26,27]
PI3K/AKT pathway	Frequently altered in HBC; *PIK3CA* mutations occur in multiple molecular subtypes	*PIK3CA* and other PI3K/AKT pathway alterations occur in CMTs	Strong molecular correspondence	[26,27]
p53/*TP53* pathway	*TP53* is frequently altered in several aggressive HBC subtypes	*TP53* alterations occur in CMTs, but reported frequency and spectrum are variable and may be lower in some cohorts	Functional conservation with genetic heterogeneity	[27,28,29,30]
Molecular subtypes	PAM50 and other molecular classifications define clinically relevant subtypes	CMT show transcriptional features overlapping selected human molecular subtypes	Partial correspondence; classifications are not interchangeable	[26,31]
EMT-related programs	EMT-associated programs are linked to invasion and aggressive disease in selected HBC subtypes	EMT activation has been identified in a CMT subgroup with basal-like features	Comparable in selected tumors	[26]
Cell-cycle regulation/INK4a/ARF	Dysregulation of cell-cycle regulators contributes to breast carcinogenesis	*INK4a/ARF*-related alterations are associated with canine mammary tumorigenesis	Functionally comparable	[32,33]
Gene-expression signatures/PEPs	Cancer-associated expression profiles can correlate with tumor aggressiveness and survival	Cancer-specific PEPs are associated with aggressiveness and can predict outcomes relevant to HBC	Selected transcriptional programs are conserved	[31,33]

Note: “Similar” indicates broadly overlapping biological or clinicopathological characteristics, whereas “comparable” indicates that a feature can be meaningfully evaluated across species but is not necessarily equivalent. “Partially conserved” indicates shared molecular or genetic mechanisms with potentially different frequencies, spectra, or biological contexts. “Identical” is intentionally avoided because direct biological equivalence between HBC and CMT cannot generally be established.

## Data Availability

No new data were created or analyzed in this study. Data sharing is not applicable to this article.
